# Correction: DARPins recognizing mTFP1 as novel reagents for *in vitro* and *in vivo* protein manipulations (doi:10.1242/bio.036749)

**DOI:** 10.1242/bio.040832

**Published:** 2018-12-15

**Authors:** M. Alessandra Vigano, Dimitri Bieli, Jonas V. Schaefer, Roman P. Jakob, Shinya Matsuda, Timm Maier, Andreas Plückthun, Markus Affolter

There were errors published in Biology Open (2018) 11, bio036749 (doi:10.1242/bio.036749).

The article title originally used the word ‘recognize’, where the word used should have been ‘recognizing’.

The correct title appears above and both the online full-text and PDF versions have been updated.

We apologise to the authors and readers for this error and any inconvenience it may have caused.

This correction does not affect the results in the article or the conclusions of this study.

