# Supplementary material for: DARPins recognize mTFP1 as novel reagents for in vitro and in vivo protein manipulations
Source: Biol Open. 2018 Sep 20;7(11):bio036749. doi: 10.1242/bio.036749 (PMC6262872; doi:10.1242/bio.036749)
Supplement: Supplementary information [file biolopen-7-036749-s1.pdf]

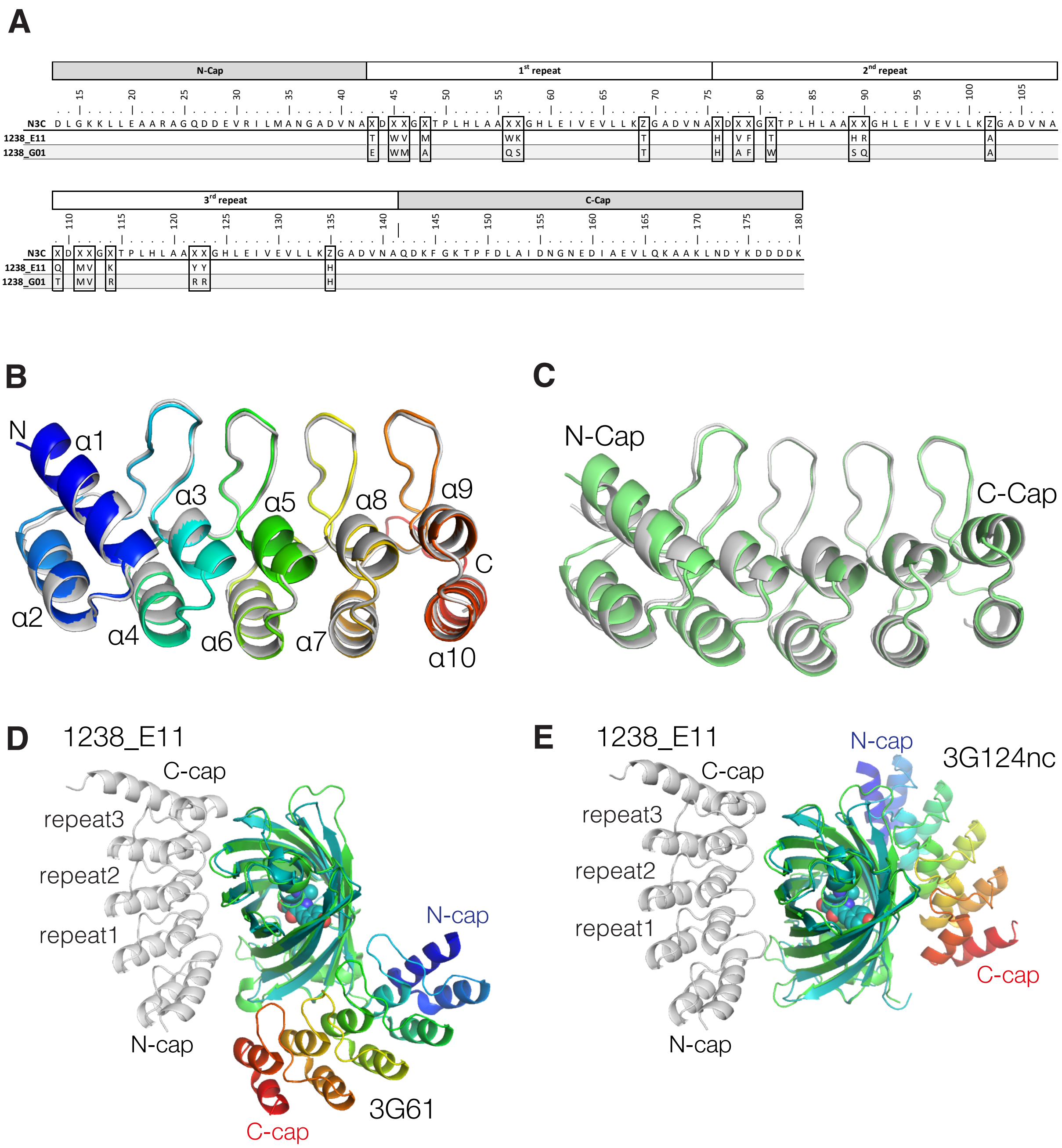

Biology Open • Supplementary information

Figure S1

Structural analysis of the mTFP1/DARPin complex.

(A) Sequences of selected DARPins. The sequences of DARPin 1238_E11 and DARPin 1238_G01 are shown. The top row indicates the consensus sequence used in the library with randomized positions indicated as X (randomization to all amino acids but Cys, Pro and Gly) and Z (randomization to Asn, His or Tyr), highlighted in black frames. In the lower lines, only residues that differ from the consensus sequence are printed. **(B)** Superposition of the DARPin 1238_G01 (grey) and DARPin 1238_E11 (rainbow-colored from N- (blue) to C-terminus (red) (C_α r.m.s.d. 0.3 Å). **(C)** Superposition of the DARPin 1238_E11 (grey) and DARPin 1238_E11 in complex with mTFP1 (lime) (C_α r.m.s.d. 0.45 Å), minor differences are only observed for the two N-Cap helices. **(D)** Superposition of the mTFP1/DARPin 1238_E11 and the GFP/ DARPin 3G61 complex (PDB ID: 5MAD) (Hansen et al., 2017). mTFP1 (teal), DARPin 1238_E11 (grey), GFP (green) and anti-GFP DARPin 3G61 (rainbow coloured, from N-terminus blue to C-terminus red) are shown in cartoon representation. The mTFP1 chromophore is shown as a sphere. **(E)** Superposition of the mTFP/DARPin 1238_E11 and the GFP/DARPin 3G124nc complex (PDB ID: 5MA8) (Hansen et al., 2017). mTFP1 (teal), DARPin 1238_E11 (grey), GFP (green) and anti-GFP DARPin 3G124nc (rainbow coloured, from N-terminus blue to C-terminus red) are shown in cartoon representation. The mTFP1 chromophore is shown as a sphere.

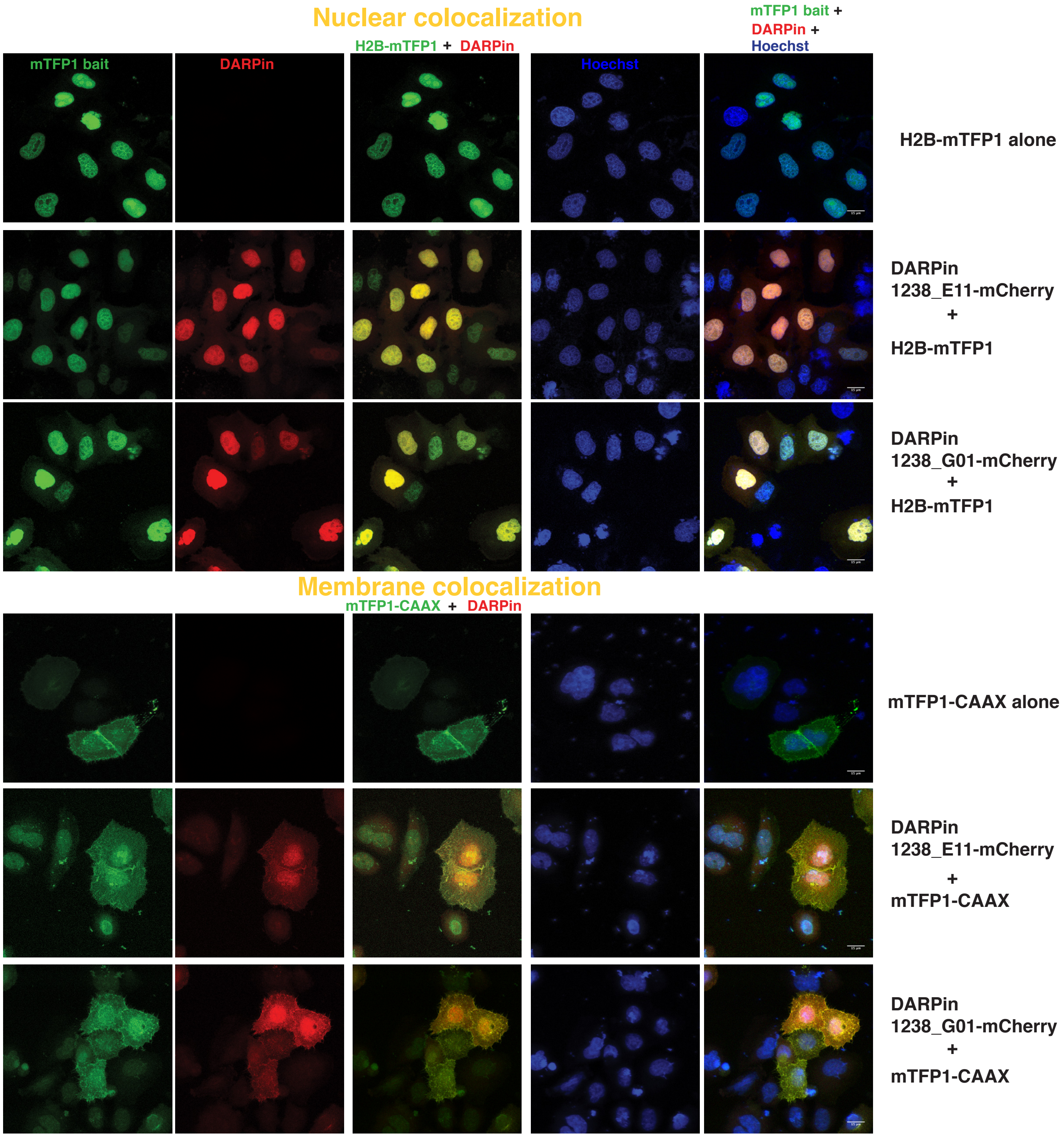

Nuclear colocalization
mTFP1 bait + DARPin + Hoechst
H2B-mTFP1 + DARPin
mTFP1 bait
DARPin
Hoechst
H2B-mTFP1 alone
DARPin 1238_E11-mCherry + H2B-mTFP1
DARPin 1238_G01-mCherry + H2B-mTFP1
Membrane colocalization
mTFP1-CAAX + DARPin
mTFP1-CAAX alone
DARPin 1238_E11-mCherry + mTFP1-CAAX
DARPin 1238_G01-mCherry + mTFP1-CAAX

Figure S2

Intracellular binding of anti-mTFP1-DARPins

Confocal images of HeLa cells transiently transfected with pH2B-mTFP1 alone (first row) or with the combination of either pH2B-mTFP1 and pCMV-DARPin 1238_E11-mCherry (second row) or pH2B-mTFP1 and pCMV-DARPin 1238_G01-mCherry (third row), respectively; pmTFP1-CAAX alone (fourth row) or the combination of either pmTFP1-CAAX and pCMV-DARPin 1238_E11-mCherry (fifth row) and or pmTFP1-CAAX and pCMV-DARPin 1238_G01-mCherry (sixth row). The first column represents the mTFP1 bait (green), the second column is the mCherry DARPin channel (red), the third column is the overlay of the first two channels, showing the nuclear colocalization of the H2B-mTFP1 bait (second and third rows) or membrane colocalization of pmTFP1_CAAX bait (fifth and sixth rows) with the respective DARPins. The fourth column represents the nuclear Hoechst staining (blue) and the fifth column is the merge of all three channels (with the scale bar in white (15 μm) on the bottom right corner). Images were taken 24 hours post transfection. Transfected constructs are indicated at the right of each row and the single channels are indicated inside the panels of the first row. The merge channels are also indicated at the top of the respective columns. The figures are from a representative experiment, performed at least three times.

Biology Open • Supplementary information

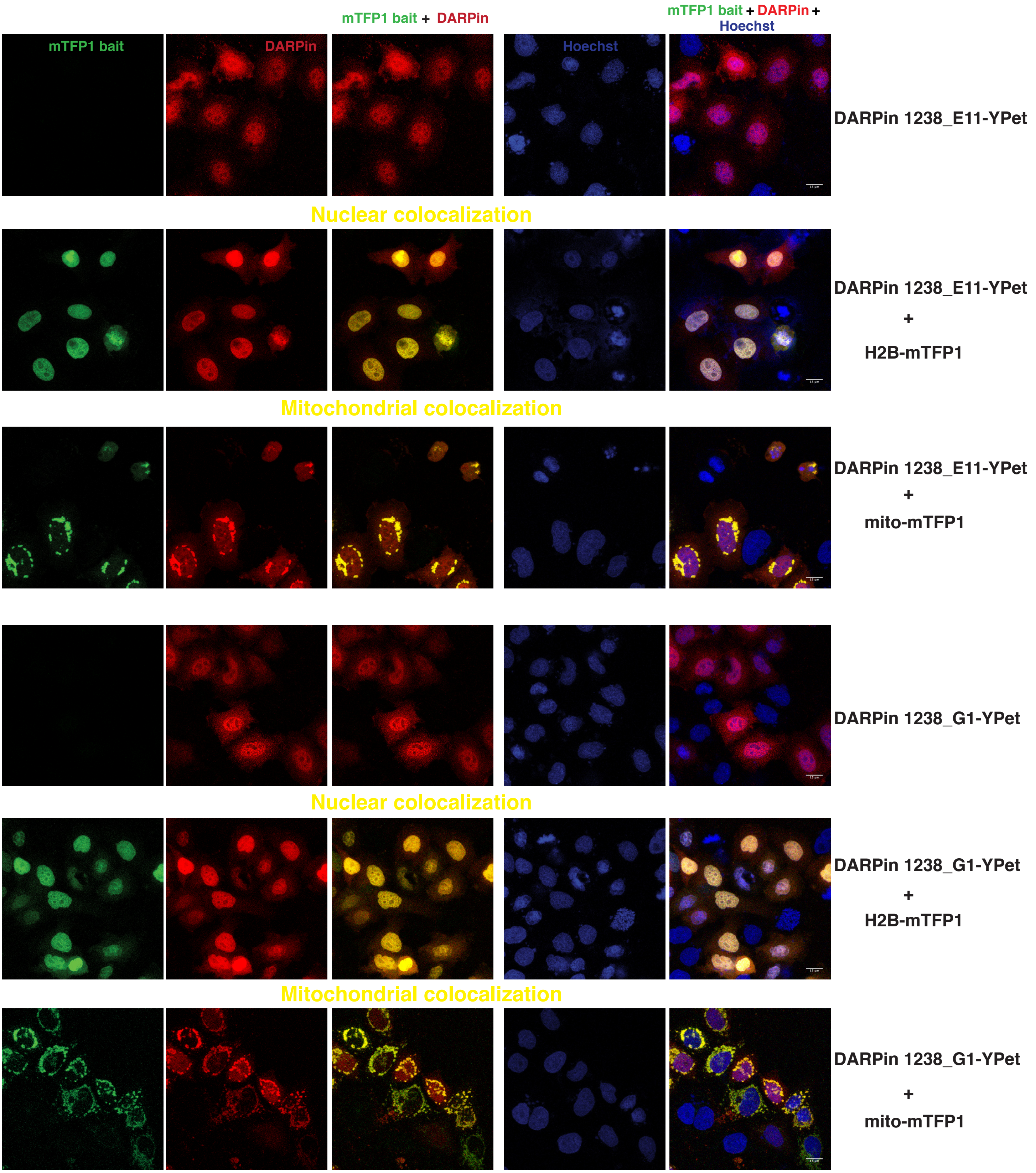
mTFP1 bait + DARPin
mTFP1 bait + DARPin + Hoechst
mTFP1 bait
DARPin
Hoechst
DARPin 1238_E11-YPet
Nuclear colocalization
DARPin 1238_E11-YPet
+
H2B-mTFP1
Mitochondrial colocalization
DARPin 1238_E11-YPet
+
mito-mTFP1
DARPin 1238_G1-YPet
Nuclear colocalization
DARPin 1238_G1-YPet
+
H2B-mTFP1
Mitochondrial colocalization
DARPin 1238_G1-YPet
+
mito-mTFP1

Figure S3

Intracellular binding of anti-mTFP1-DARPins-YPet

Confocal images of HeLa cells transiently transfected with pCMV-DARPin 1238_E11_YPet alone (first row) or in combination with pH2B-mTFP1 (second row) or mito-mTFP1 (third row). The same transfection scheme is presented for the DARPin 1238_G01-YPet alone (fourth row) or in combination with H2B-mTFP1 (fifth row) or mito-mTFP1 (sixth row). The first column represents the mTFP1 channel (green), the second column is the YPet channel (red), the third column is the overlay of the two channels, showing the colocalization of the DARPins-YPet fusion to the different mTFP1 baits, the fourth column represents the nuclear Hoechst staining (blue) and the fifth column is the merge of all three channels (with the scale bar in white (15 μm) on the bottom right corner). Images were taken 24 hours post transfection. Transfected constructs are indicated at the right of each row and the single channels are indicated inside the panels of the first row. The merge channels are also indicated at the top of the respective columns. The figures are from a representative experiment, performed at least three times.

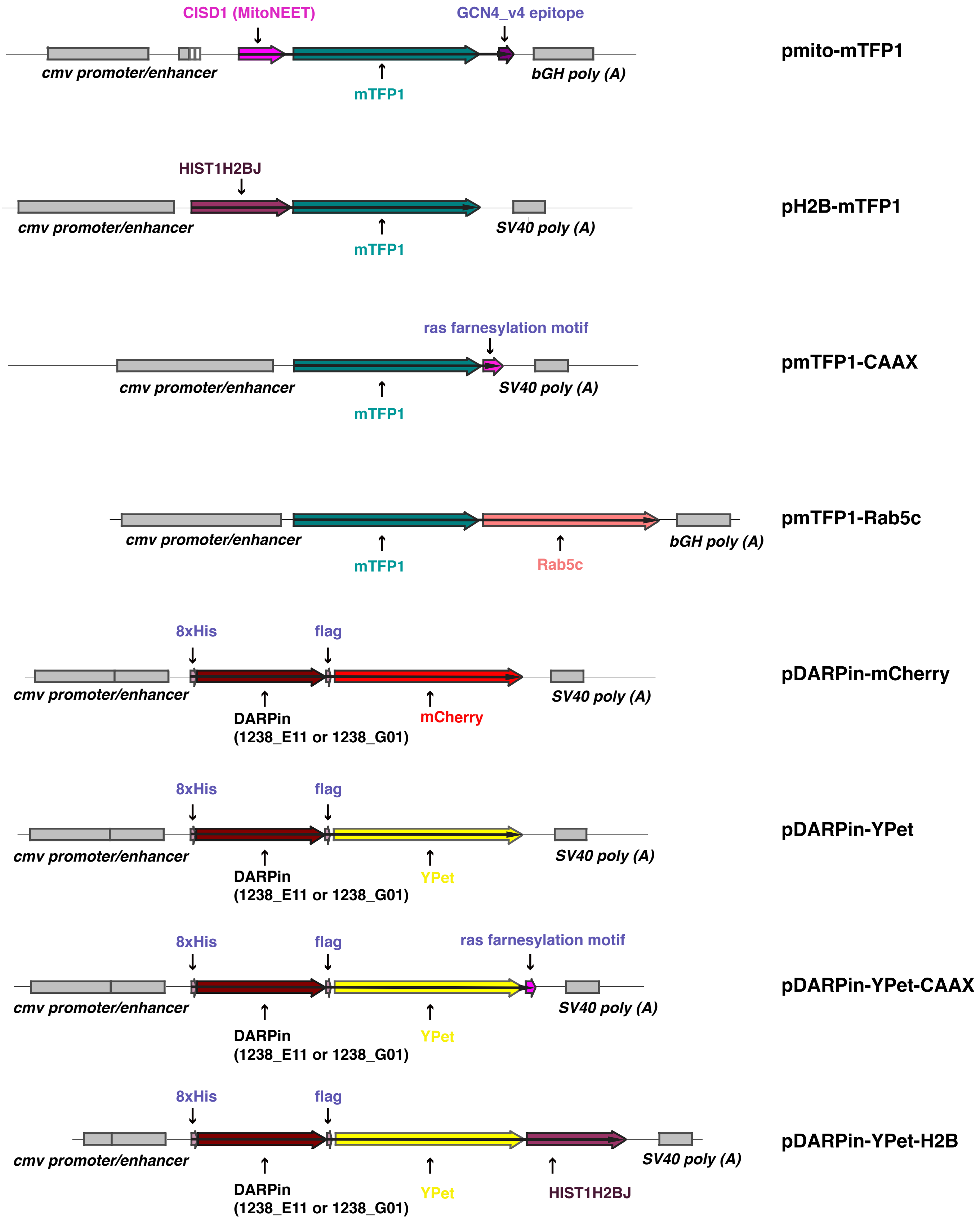

Biology Open • Supplementary information

Figure S4

Schematic representation of the fusion constructs

The transcriptional elements (enhancer, promoter and poly (A) adenylation) of the different mammalian expression vectors are depicted as grey filled boxes. The different protein coding modules are represented as coloured block arrows, while the resulting fusion protein is depicted as a solid black arrow inside the modules. Full maps and sequences are available upon request.

Table S1. Statistics on diffraction data and refinement of DARPin 1238_E11, DARPin 1238_G01, and mTFP1/DARPin 1238_E11 complexes.

	mTFP1/ DARPin 1238_E11 ($P6_522$)	mTFP1/ DARPin 1238_E11 (C2)	DARPin 1238_E11 ($P2_1$)	DARPin 1238_G01 ($P2_12_12_1$)	DARPin 1238_G01 (I4)
PDB Identifier	6FP7	6FP8	6FP9	6FPA	6FPB
Wavelength (Å)	0.99986	0.99986	1.00003	0.99986	0.99986
Resolution range (Å)	45.45 - 1.58 (1.67 - 1.58)*	39.61 - 1.85 (1.97 – 1.85)*	48.50 – 2.10 (2.16 – 2.10)*	44.58 - 1.58 (1.68 - 1.58)*	46.80 – 1.62 (1.72 - 1.62)*
Space group	P 6_5 2 2	C2	P 2_1	P 2_1 2_1 2_1	I4
Unit cell	99.1 99.1 214.6	136.0 75.0 48.3	51.7 91.6 54.4	38.0 89.2 103.8	66.2 66.2 77.9
aα, β, γ (°)	90 90 120	90 90.7 90	90 116.9 90	90 90 90	90 90 90
Total reflections	2190376 (327328)	187610 (28854)	94272 (5694)	426339 (39829)	96928 (9216)
Unique reflections	161477 (25309)	78111 (12129)	25698 (1843)	92906 (9440)	41912 (4096)
Multiplicity	13.5 (12.9)	2.4 (2.3)	3.7 (3.1)	4.6 (4.2)	2.2 (2.2)
Completeness (%)	99.3 (96.4)	96.6 (93.1)	99.4 (85.8)	99.2 (97.0)	99.3 (97.1)
Mean I/sigma(I)	13.6 (1.3)	11.2 (1.7)	7.7 (2.1)	14.1 (1.2)	11.5 (1.3)
Wilson B-factor	22.1	29.8	31.1	21.6	30.8
R-meas	0.144 (1.98)	0.069 (0.730)	0.273 (1.54)	0.078 (1.19)	0.066 (0.935)
CC1/2	0.999 (0.572)	0.998 (0.652)	0.975 (0.39)	0.999 (0.51)	0.998 (0.746)
R-work	0.148 (0.272)	0.149 (0.237)	0.162 (0.237)	0.146 (0.306)	0.152 (0.319)
R-free	0.164 (0.287)	0.174 (0.251)	0.205 (0.243)	0.180 (0.310)	0.175 (0.338)
Number of atoms	6929	6562	5338	5450	2593
water	732	444	475	589	197
Protein residues	386	384	334	321	159
RMS(bonds)	0.005	0.012	0.008	0.006	0.009
RMS(angles)	0.92	1.20	0.81	0.86	1.02
Ramachandran favored (%)	99.0	99.2	99.4	99.7	98.1
Ramachandran outliers (%)	0	0	0	0	0
Clashscore	1.13	1.62	1.18	0.62	1.67
Average B-factor	35.0	40.0	24.0	29.0	47.0

*The values in parentheses correspond to the highest resolution shell.

Table S2

Click here to Download Table S2
